# Obstructive pneumonia owing to migration of a Teflon pledget at 8 years after surgery for a pulmonary carcinoid tumor: a case report

**DOI:** 10.1186/s40792-019-0734-2

**Published:** 2019-10-29

**Authors:** Hikaru Watanabe, Kohei Abe, Naoki Kanauchi

**Affiliations:** Department of General Thoracic Surgery, Nihonkai General Hospital, 30, Akiho-cho, Sakata, Yamagata 981-8501 Japan

**Keywords:** Late complication, Non-absorbable suture material, Pulmonary resection, Lung carcinoid tumor

## Abstract

**Background:**

It is uncommon for a bronchial stump-related complication to develop during the remote postoperative period in a case of obstructive pneumonia owing to migration of the suture material. Here, we describe a case of bronchial obstructive pneumonia that developed owing to migration of the suture material in the airway 8 years after pulmonary resection.

**Case presentation:**

A 34-year-old woman underwent left lower lobectomy for a pulmonary carcinoid tumor (pT1bN0M0-stage IA) in 2010. She experienced obstructive pneumonia, and chest computed tomography revealed a mass protruding from the bronchial stump to the bronchial lumen in 2018. After treatment for pneumonia, flexible bronchoscopy showed the presence of a fibrous suture material (Teflon pledget) completely obstructing the left second carina. A week later, the Teflon pledget obstructing the bronchial lumen was removed using a flexible bronchoscope with the patient under general anesthesia. The procedure was completed without removing the small amount of granulation tissue because the bronchial lumen opened after removing the Teflon pledget. She has remained asymptomatic for 1 year after removal.

**Conclusions:**

In this case, the complication of obstructive pneumonia developed owing to migration of the non-absorbable suture materials used to suture the bronchial stump. Bronchoscopic management of this rare complication comprised endobronchial removal with the patient under general anesthesia. Given our experience with this case, we believe that such conservative management should allow for excellent results in most instances and avoid the need for reoperation.

## Background

Bronchial fistula is a critical complication after pulmonary resection. Different techniques of reinforcing the bronchial stump to prevent this complication have been described, and the incidence of this complication has decreased in recent decades. Pledget sutures have been suggested for this purpose but have the potential to become infected and erode into the bronchus years after pulmonary resection. For example, there are a small number of reported cases of intrabronchial granuloma formation owing to the suture material used in pulmonary resection during the remote postoperative period [1, 2]. However, it is extremely rare to observe the exposure of suture material itself in the bronchial lumen during the remote postoperative period [2]. Herein, we describe a case of obstructive pneumonia owing to the migration of the suture material used for bronchial stump closure that became exposed in the bronchial lumen 8 years after surgery for a lung carcinoid tumor.

## Case presentation

A 34-year-old woman underwent left lower lobectomy for a pulmonary carcinoid tumor (pT1bN0M0-stage IA). Postoperatively, there was no pulmonary fistula or bronchial stump fistula, and her clinical course was favorable. She had been doing well for 8 years after completing therapy until 42 years of age when she visited a clinic with the chief complaints of fever and sputum. Chest plain radiography showed left-sided pneumonia, for which antibiotic treatment was initiated. Although the blood test result showed improvement of inflammatory markers, chest plain radiography showed no improvement in pneumonia; therefore, she was referred to our hospital.

**Fig. 1:**
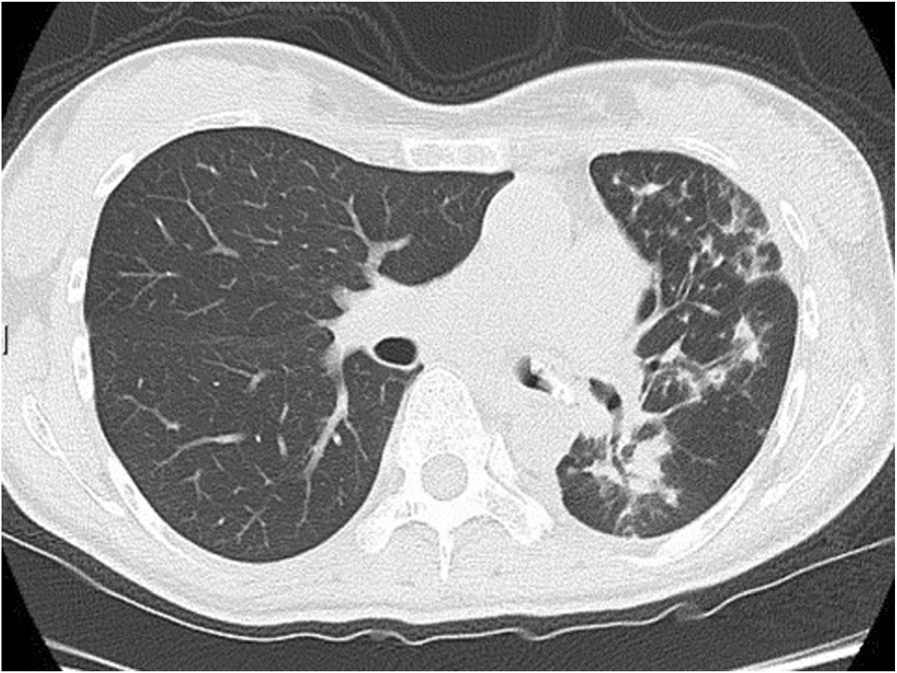
Axial chest computed tomography (CT) images at admission show a mass surrounding the patient’s left lower bronchus stump with endobronchial extension causing significant obstruction of the left upper bronchus

**Fig. 2:**
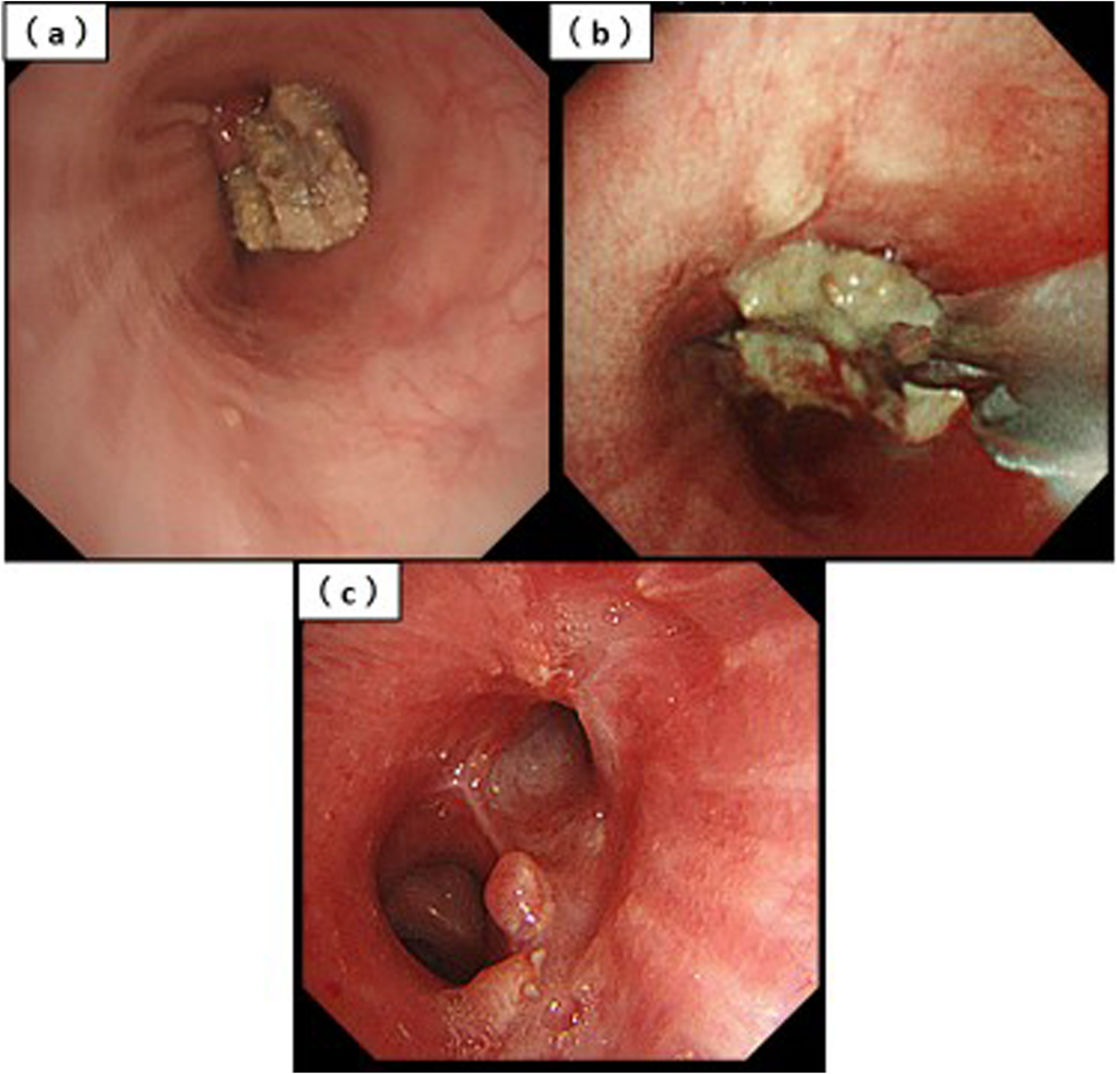
Bronchoscopic findings. **a** Bronchoscopy at admission shows a Teflon pledget in the bronchial lumen. **b** The Teflon pledget obstructing the bronchial lumen is removed using a flexible bronchoscope with the patient under general anesthesia. **c** Bronchoscopy at 1 month after treatment shows the bronchial lumen is still open

Chest computed tomography (CT) showed a tumorous lesion nearly obstructing the bronchial lumen and the extension of pneumonia into the residual left lung (Fig. 1). Recurrence of the bronchial stump was suspected based on her medical history. Subsequently, flexible bronchoscopy was performed and showed the presence of a mass, a fibrous suture material, that was completely obstructing the left second carina. Results of the biopsy revealed only fiber without evidence of malignancy (Fig. 2a). The sputum, blood, granuloma, and biopsy fiber cultures were negative.

Considering the risk of hemoptysis and bronchial stump fistula, the suture material obstructing the bronchial lumen was removed using a flexible bronchoscope with the patient under general anesthesia 1 week later (Fig. 2b). Little granulation tissue was observed at the site of the removal. Although there was some minor oozing of blood from the same site, hemostasis was achieved with argon plasma coagulation therapy. The procedure was completed without removing the small amount of granulation tissue because the bronchial lumen opened after removal of the suture material.

According to a copy of the 2010 operating report, the bronchial stump was sutured with five stitches using an absorbable monofilament suture (4–0 polydioxanone suture [PDS]). However, the left lower bronchus stump had hypertonicity. To decrease the tension at the suture site, a 4–0 PDS attached to the Teflon pledget (CROWN JUN, size 6 mm × 10 mm) with two needles was placed in the central part of the stump. This procedure and our bronchoscopic findings indicated that the bronchial obstruction was secondary to the endobronchial migration of the Teflon pledget that had eroded through the bronchial wall.

**Fig. 3:**
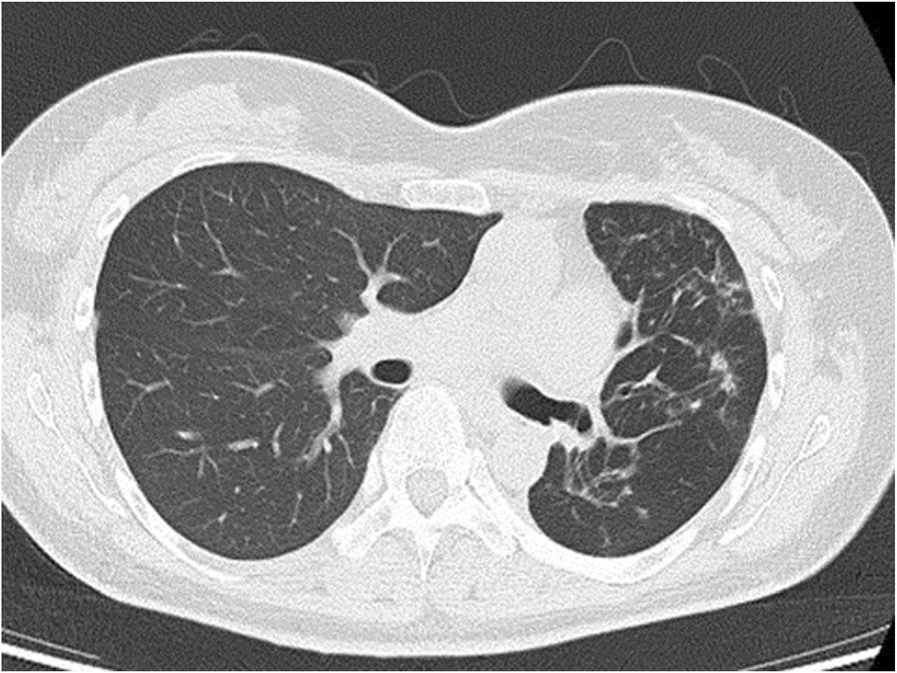
Axial chest CT reveals that the left upper bronchus obstruction was released 4 months after the procedure

At 1 month after removal, bronchoscopy showed pedunculated granulation tissue, the biopsy result showed no malignant findings, and the bronchial lumen was still open (Fig. 2c). At 4 months after removal, CT showed disappearance of the tumorous lesion protruding into the bronchial lumen and disappearance of pneumonia (Fig. 3).

The patient’s clinical course after the procedure was favorable. At 1 year after Teflon pledget removal, there were no recurrences of the pulmonary carcinoid tumor or obstructive pneumonia.

## Discussions

In thoracic surgery, one of the important concerns that has led to an insistent demand for suture protection is bronchopleural fistula. Risk factors for the development of bronchopleural fistula include advanced cancer stage, preoperative chemotherapy or radiation therapy or both, previous steroid therapy, residual carcinomatous stump, postoperative mechanical ventilation, chronic obstructive pulmonary disease, diabetes mellitus, and malnutrition [3, 4]. The use of Teflon pledgets has been reported to reinforce the bronchial stump and potentially reduce the incidence of bronchopleural fistula, especially in patients who are at an increased risk [5]. Nevertheless, a case of bronchial obstruction owing to the erosion of Teflon pledgets into the airway has been reported previously [2].

Wound healing of the bronchial stump is characterized by granulation tissue formation with angiogenesis and proliferation of fibroblast cells. During this process, a foreign substance such as a suture can become buried in the connective tissue. Healing of the bronchial stump is not directly attributable to healing of the suture site, but it is achieved secondarily when the bronchial stump is covered with peribronchial tissue. The outer surface of the bronchial stump is covered by connective tissue within 2 weeks, and complete healing with mature fibrous tissue is achieved within 4 weeks [6]. In accordance with this mechanism, suture material for bronchial stump closure is buried in the connective tissue or fibrous tissue but not exposed on the pleural surface. However, there have been cases of infection owing to a foreign substance such as a suture or chronic infection that led to the development of refractory granuloma [7]. The reported suture material-related complications occurring at least 1 year postoperatively include granuloma, hemoptysis, infection, stump fistula, and chronic necrotizing pulmonary aspergillosis [1, 2]. No study other than the present one has reported exposure of the suture material in the bronchial stump at 8 years after lobectomy.

The absorbable suture, which was used in the present case, is safe because the tensile strength is maintained for 2 weeks until the anastomosis site and bronchial stump are covered by connective tissue, although there is a concern for suture failure owing to an early decrease in tensile strength [8]. In the present case, there had been no air leakage from the bronchial stump. Therefore, considering the healing mechanism of the bronchial stump, it might have been necessary to use the Teflon pledget for decreasing tension. However, with that as a cause, our patient developed this complication after lobectomy. The previously reported suture materials related to complications were mostly non-absorbable materials [2], and the suture material used in the present case was a Teflon pledget, a non-absorbable material.

To prevent late complications, it is desirable to use biological materials such as fat pad, muscle fascia, and pericardium. However, in cases where the wound healing process is not compromised, late complications can be prevented even with non-absorbable suture materials. Regarding the mechanism of translocation of the suture material from the pleural space to the bronchial lumen in the present case, it was hypothesized that the suture with the pledget remaining in the pleural space had been buried in the submucosa of the bronchial wall during the wound healing process after the bronchus was cut. However, it is difficult to prove this hypothesis.

## Conclusions

We described about a patient who developed a late complication owing to non-absorbable suture material at 8 years after lobectomy. The results indicated that if a non-absorbable suture material has been used, surgeons should be aware of the risk of exposure of suture material in the bronchial stump during the remote postoperative period, although it is extremely rare. In such cases, the pleural side of the suture material is considered to be covered with connective tissue and fibrous tissue according to the mechanism of wound healing. Therefore, if the complication occurs, it is possible to rapidly remove the foreign material endoscopically.

## Data Availability

The data are not available for public access because of patient privacy concerns but are available from the corresponding author on reasonable request.

## Abbreviations

CT: Computed tomography
PDS: Polydioxanone suture

## References

[1] Aboudara M, Krimsky W, Harley D (2012). Teflon haemoptysis. BMJ Case Rep.

[2] Precht LM, Vallières E (2008). Bronchial obstruction due to Teflon pledgets migration 13 years after lobectomy. Ann Thorac Surg.

[3] Sirbu H, Busch T, Aleksic I, Schreiner W, Oster O, Dalichau H (2001). Bronchopleural fistula in the surgery of non-small cell lung cancer: incidence, risk factor, and management. Ann Thoracic Cardiovasc Surg.

[4] Algar FJ, Alvarez A, Aranda JL, Salvatierra A, Baamonde C, Lopez-Pujol FJ (2001). Prediction of early bronchopleural fistula after pneumonectomy: a multivariate analysis. Ann Thorac Surg.

[5] Sonobe M, Nakagawa M, Ichinose M, Ikegami N, Nagasawa M, Shindo T (2000). Analysis of risk factor in bronchopleural fistula after pulmonary resection for primary lung cancer. Eur J Cardiothorac Surg.

[6] Rienhoff WF, Gannon J, Sherman I (1942). Closure of the bronchus following total pneumonectomy. Ann Surg.

[7] Chouabe S, Perdu D, Deslée G, Milosevic D, Marque E, Lebargy F (2002). Endobronchial actinomycosis associated with foreign body: four cases and a review of the literature. Chest.

[8] Muramatsu T, Ohata M, Iida M, Ohmori K, Irako M, Kitamura K (1992). Tracheal anastomosis with new synthetic monofilament absorbable suture. J Jpn Assoc Chest Surg.

